# Participatory approaches for raising awareness among subsistence farmers in Tanzania about the spread of insecticide resistance in malaria vectors and the possible link to improper agricultural pesticide use

**DOI:** 10.1186/s12936-022-04289-1

**Published:** 2022-09-30

**Authors:** Nancy Stephen Matowo, Marcel Tanner, Benigni Alfred Temba, Marceline Finda, Yeromin Paul Mlacha, Jürg Utzinger, Fredros Oketch Okumu

**Affiliations:** 1 Environmental and Ecological Sciences Department, Ifakara Health Institute, Ifakara, Tanzania; 2 Swiss Tropical and Public Health Institute, Allschwil, Switzerland; 3 University of Basel, Basel, Switzerland; 4 Department of Disease Control, London School of Hygiene and Tropical Medicine, London, UK; 5 Department of Veterinary Physiology, Pharmacology and Toxicology, Sokoine University of Agriculture, Morogoro, Tanzania; 6 School of Public Health, Faculty of Health Sciences, University of the Witwatersrand, Johannesburg, South Africa; 7 Institute of Biodiversity, Animal Health and Comparative Medicine, University of Glasgow, Glasgow, UK

**Keywords:** Agricultural pesticides, Agricultural practices, *Anopheles* mosquitoes, Crop pests, Insecticide resistance, Malaria, Participatory learning, Tanzania

## Abstract

**Background:**

Insecticide resistance is a key barrier to long-term malaria control, and it may be exacerbated by poor agricultural pesticide use. Current practices, however, do not link public health and agricultural pesticide use. This study investigated the perspectives of farmers and other stakeholders regarding the integration of agricultural and public health measures to address resistance. Additionally, the feasibility of participatory workshops to increase the farmers’ understanding and participation in pesticide stewardship was assessed.

**Methods:**

Four themes were investigated: pesticide awareness, practices, and opinions of; insecticide resistance in malaria vectors; the effectiveness of current malaria prevention tools; and the links between agricultural and public health pesticide usage. Participatory workshops and field training were held with entomologists, farmers, and agricultural specialists, focusing on agro-ecosystem practices related to pest control; and local farmers were involved in live-testing for insecticides resistance of local Anopheles mosquitoes.

**Results:**

Most farmers (94%) considered pesticides effective, and nearly half of them (n = 198, 46.4%) could identify and name crop pests and diseases, mostly using local names. Three quarters were unaware of mosquito larvae in their fields, and only 7% considered their fields as potential sources of mosquitoes. Two thirds were uninformed of any effects that agricultural pesticides may have on mosquitoes, and three quarters had never heard of resistance in malaria mosquitoes. Experts from various sectors acknowledged that agricultural pesticides might impact malaria control through increasing resistance. They did, however, emphasize the importance of crop protection and advocated for the use of pesticides sparingly and non-chemical approaches. Farmers learnt how to discriminate between malaria vectors and non-vectors, identify agricultural pests and diseases, choose and use pesticides effectively, and conduct resistance tests during the participatory workshops.

**Conclusion:**

This study emphasizes the significance of enhancing subsistence farmers’ awareness of mosquito ecology as well as merging public health and agricultural pest management measures. Participatory techniques have the potential to raise stakeholder awareness and engagement, resulting in more effective resistance management.

## Background

In sub-Saharan Africa, malaria prevention relies primarily on long-lasting insecticidal nets (LLINs) and indoor residual spraying (IRS) [1, 2]. In Tanzania, LLINs are distributed throughout the country [3, 4], whereas IRS is primarily used in the north-western regions of the country around Lake Victoria and on the islands of Zanzibar [4, 5]. Despite considerable progress made over the past 20 years, the burden of malaria in Tanzania remains high. In 2017, the national prevalence among children under the age of five years was 7.3% [6]. Recent data suggest considerable spatial heterogeneity in malaria transmission [7, 8]. Furthermore, the continuing COVID-19 pandemic might stall and revert prior successes in malaria control [9, 10].

Persistent malaria transmission is partly attributed to the recent changes in malaria vector populations, notably behavioural or physiologically resistance after prolonged use of LLINs and IRS [11, 12]. Indeed, both the strength and distribution of resistance have increased in Africa [13], and most countries had to change the classes of insecticides over time for effective vector control [14–17]. While LLINs continue to rely predominantly on pyrethroids, IRS now includes insecticide classes previously used in agriculture, notably organophosphates and neonicotinoids [18].

Agriculture is critical to the economies and livelihoods of the majority of African countries. However, the resulting agro-ecosystems provide favourable environments for mosquito vectors to breed. Besides, crop pest management relies on synthetic pesticides, which are frequently the same classes as those used in public health [19]. According to Tanzania estimates, a large share, 81% of synthetic pesticides are used for agricultural purpose by small-scale farmers to protect crop from pests and diseases [20]. Unfortunately, selection pressures associated with widespread agricultural pesticides may influence the evolution of insecticide resistance in malaria vectors [21], as farmers are frequently unaware of potential impact their actions on disease transmission. A recent study in rural Tanzania demonstrated overlap between insecticide classes used in public health and agriculture in an environment where agricultural pesticide use was largely uncontrolled [22]. These challenges corroborate previous findings that small-holder farming communities may face the highest risk of malaria as a result of occupational exposures [23–26], cultural and behavioural practices (e.g., migratory farming practices) [27, 28], and limited access to malaria prevention and prompt treatment services [27, 28]. Regrettably, the World Health Organization (WHO) global action plan to control the spread of insecticide resistance in malaria vectors, did not include practical recommendations for addressing gaps in agricultural practices related to malaria control [29]. Current pest management practices do not consider the relationship between public health and agricultural pesticide use.

Tanzania’s current National Malaria Strategic Plan 2014–2020 emphasizes the critical role of inter-sectoral coordination in malaria vector control [4]. The double-edged contributions of agriculture in food production and promoting resistance in disease vectors, however, must be recognized and implemented [4]. While community members are considered primary partners in vector control but they are not adequately empowered or involved in the implementation of resistance management programmes [4]. However, significant collaboration and participation are required to improve malaria control in agriculturally dominated areas, particularly irrigated rice farming [30, 31].

The purpose of this study was to explore the opinions of key stakeholders on potential approaches for integrating agricultural and public health practices to address resistance in malaria vectors. Additionally, the feasibility of participatory workshops for increasing awareness and participation of subsistence farmers in pesticide stewardship was determined. The study began with an assessment of current pest management practices, public awareness of the connection between agriculture and malaria, and perceptions of insecticide resistance in malaria vectors. It also discussed the perspectives of key stakeholders on the need for, and potential approaches to integrating agricultural practices into pests and disease vectors management strategies.

## Methods

### Study area

**Fig. 1:**
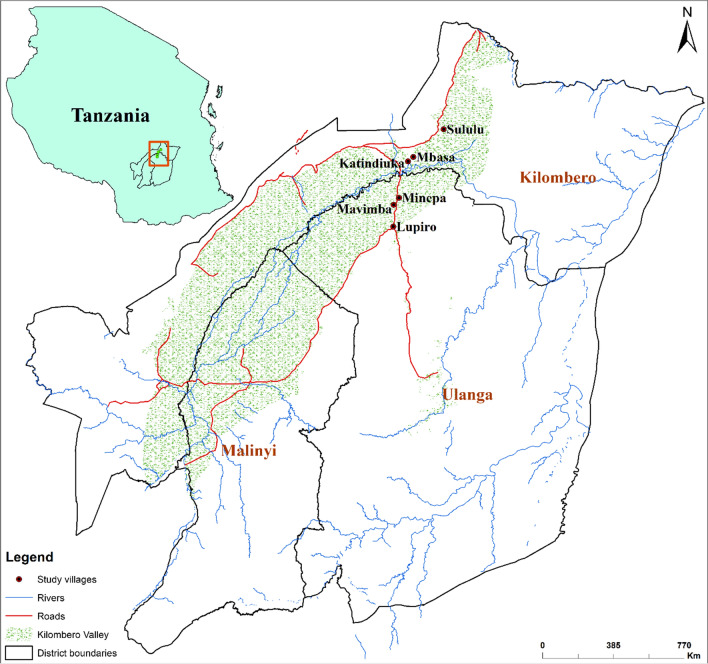
Map showing the study wards in the districts of Kilombero and Ulanga in the south-eastern parts of Tanzania, where the current investigation was carried out

The study was carried out in six wards (i.e., Katindiuka, Lupiro, Mavimba, Mbasa, Minepa, and Sululu) in the districts of Kilombero and Ulanga in Tanzania’s south-eastern region, rising 120–350 m above sea level on the flood plains of the Kilombero River valley (Fig. 1). Rice farming and fishing are the primary sources of food and income. During the dry season, rice production is sustained by an irrigated system locally known as “Ngapa”, which also supports local mosquito populations [27]. There is a stable transmission of *Plasmodium falciparum* malaria transmission throughout the year [32], mediated primarily by *Anopheles arabiensis* and *Anopheles funestus sensu stricto* (*s.s*.) [33]. In the study area, there is also a high density of *Culex* mosquitoes, generating significant biting nuisances [34]. Malaria control is mainly by LLINs treated with pyrethroids (mainly deltamethrin and permethrin) [3]. In this setting, the farmers also use a variety of pesticides (i.e., pyrethroids, carbamates, neonicotinoids, and organophosphates) to boost crop yields [22]. There is evidence, however, of mosquito resistance to the pyrethroids, DDT, and bendiocarb, which is most likely mediated by metabolic enzymes [33, 35]. The study was conducted at different time period between 2016 and 2018.

### Study design and data collection

This study used an exploratory sequential mixed methods design. This involved in-depth interviews with 57 small-holder farmers, followed by a questionnaire survey enrolling 427 farmers. In-depth interviews were conducted with nine key informants from the public health, agriculture, and environmental sectors. Key findings were used to develop participatory workshops and training sessions with farmers and agricultural experts to better understand their perspective on the interactions between agricultural practices and mosquito control.

#### In-depth interviews

In-depth interviews were carried out with 57 farmers (30 from Ulanga district and 27 from Kilombero district). The interview guide was developed in English, translated into Kiswahili and used in the latter language. For confidentiality of respondents, unique identification numbers were assigned to match audio-recorded data. Open-ended questions with sub-questions for probing were administered to the farmers who participated in the study. The interview questions were classified and focused on six themes, namely (i) knowledge on pests, pest management practices, and knowledge of alternative non-chemical pest management; (ii) general knowledge on malaria, mosquito ecology, and linkage with agricultural practices; (iii) opinions on malaria trends in their village; (iv) benefits and challenges of current malaria control methods; (v) views on agrochemical usage and perceived effects of the chemicals on malaria vectors; and (vi) awareness and views on insecticide resistance in mosquitoes. All of the interviews were audio-recorded, and field notes were taken to complement the interviews.

#### Survey questionnaire

Findings from the qualitative study guided development of questions for quantitative research. In total, 427 farmers were chosen at random and consented to participate in the survey. The questionnaire was translated and administered in Kiswahili, using electronic forms on a free-access software programme, KoBoToolbox [36]. The questionnaire survey covered similar topics, but with adaptations based on preliminary findings of the aforementioned in-depth interviews. Data triangulation was conducted by integrating, interpreting, and comparing findings from both methods to improve understanding of the research questions.

#### Key informant interviews

In-depth interviews were conducted with nine purposively selected stakeholders from public health, agriculture, and environmental sectors, who had a direct or indirect impact on malaria and vector control. The key informants included two ward agricultural officers, one ward veterinary officer, one acting director general of the government institute, two university lecturers, two research scientists with leadership positions at departmental level, and two malaria control programme managers. The organizations represented include (i) the National Malaria Control Programme that is managed by the Ministry of Health; (ii) the National Institute for Medical Research (NIMR); (iii) the National Environment Management Council (NEMC); (iv) the Tropical Pesticides Research Institutes (TPRI) under the Ministry of Agriculture; (v) the Muhimbili University of Health and Allied Sciences (MUHAS); and (vi) the Sokoine University of Agriculture (SUA). These interviews explored participants’ views regarding (i) malaria vector control interventions, progress, and challenges; (ii) linkages between agricultural practices and malaria control; (iii) effects of agricultural pesticides in resistance development in malaria mosquitoes; (iv) government policies and guidelines for regulating the use of agricultural pesticides; and (v) integrated vector management and inter-sectoral collaborations. The interview’s responses were audio-recorded, and specific notes were taken.

#### Participatory workshops and practical sessions with farmers and agricultural experts

Four field-based participatory workshops and three on-site field visits were conducted for the researchers, farming communities, and agricultural and veterinary officers to meet and share knowledge, experiences, and challenges related to pesticides usage practices and crop pest management. These activities also provided opportunities to engage the farming communities and agricultural experts in understanding interactions between agricultural practices and mosquito control. A total of 26 farmers and three ward agricultural officers participated in the first two workshop sessions and practical learning in the farms, while 22 farmers, two ward agricultural officers, and one ward veterinary officer were involved in the last two sessions of the workshops and field visits.

The participatory workshops were partly motivated by and curriculum developed based on the recommendations raised by the participants during earlier data collection (Table 1). Video filming, photographs, and note-taking guided the collection of relevant information generated during workshop discussions and practical field visits. During the workshops, researchers presented summary feedback of previous investigations of resistance in local malaria vectors. They also presented a summary table showing WHO-approved insecticides for malaria vector control. At the same meetings, the researchers, together with the agricultural and veterinary experts, displayed and described common pesticides used and agricultural insecticides available in nearby agrovet stores. The agrovet stores are shops where they sell agricultural and veterinary supplies including pesticides. In this study, the farmers were invited to share their experiences on use of agricultural pesticides and any challenges encountered. Similarities between active ingredients in both public health insecticides and agricultural pesticides were discussed and clarified. The concept of insecticide resistance in mosquito was explained in lay terms using simulated pictures on a video presentation. Additional open discussions were conducted with the farmers and agricultural experts, focusing on the same topics as in the aforementioned interviews.

**Table 1:** Topics and learning objectives pursued during the participatory workshops and practical field sessions during the study

Category	Workshop/topic		Learning objective
Basic knowledge and skills on malaria mosquitoes, breeding sites, and control approaches	1	Known associations between agricultural practices and mosquitoes	• Participants should be able to identify potential aquatic habitats for mosquitoes in or near their farms • Participants should understand the effects of agricultural practices on mosquitoes
	2	Sampling and identification of mosquito larvae and pupae	• Participants should be able to sample larvae and pupae from habitats • Participants should be able to identify and distinguish *Anopheles* from other larvae • Participants should be able to distinguish between male and female mosquitoes • Participants should be able to identify adult *Anopheles* and non-*Anopheles* mosquitoes
	3	Larval source management through improved agricultural practices	Farmers should be able to identify and destruct (clearing of ditches, soil filling, and draining) of suitable aquatic mosquito breeding sites linked to agricultural practices
Agricultural pesticides, public health pesticides, and malaria mosquitoes	4	Exploring the linkage between public health insecticides and agricultural insecticides	• Encourage farmers to share their farming experiences, including demonstrating the use of agricultural pesticides • Experts to share the experiences with the farmers on the performance of various agricultural pesticides • Researchers to demonstrate to the farmers and experts on insecticides used in public health against malaria and its link to agricultural insecticides • Farmers to be able to read labels, understand agricultural pesticides and its chemical ingredients before spraying
		Demonstrating the effects of agricultural pesticides on malaria vectors	Farmers should be able to understand the possible link of agricultural pesticides sprayed in the farms and their consequences, such as insecticide resistance in mosquito vectors
Management of crop pests and diseases	5	Collection and identification of common crop pests at the field	• Farmers should be able to identify individual common crop pests and diseases • Farmers should understand appropriate insecticides to spray against particular pests and diseases
Other options for crop pests management	6	Alternative crop pests and diseases management practices other than using agricultural pesticides	• Farmers should be able to suggest alternative pest-, disease-, and weed-control in crops other than using pesticides • Agricultural pesticides as the last option in controlling pests and diseases in crops
Good agricultural practices for pesticide management	7	Proper storage and disposals of agricultural pesticides	Experts and farmers should demonstrate good and safe agricultural practices for handling, keeping, or disposing of leftover pesticides, and empting containers

Additional sessions were organized where participants conducted practical field activities and discussions on the interactions between agro-ecosystem and mosquito ecology. These included (i) sampling and identification of anopheline and culicine mosquito larvae; (ii) direct observations of different life-cycle stages of mosquitoes; (iii) demonstration of farming practices aimed at minimizing mosquito breeding; (iv) sampling and identification of adult crop pests and predators; (vi) demonstrations of proper handling, storage, and disposal of agricultural pesticides; and (vii) observations of non-insecticidal methods against crop pests, diseases, and weeds. Pesticides commonly used by the farmers were borrowed from the local agrovet stores and used for demonstration. These including Karate 5EC (lamba-cyahalothrin), Duduba 450EC (cypermethrin and chlorpyrifos), and Actellic 50EC® (pirimiphos-methyl).

#### Participatory testing of insecticide susceptibility in local malaria vectors

During the participatory sessions, individuals were co-opted to participate in investigations of phenotypic resistance of malaria vectors. After initial training, the tests were pursued jointly with farmers, agricultural experts, and researchers, using female *An. arabiensis* mosquitoes raised from larvae that had been collected by the same farmers from their farms. The susceptibility tests were carried out in accordance with standard WHO guidelines [37]. The efficacy of insecticide-impregnated test papers was first validated against a laboratory-reared susceptible strain of *Anopheles gambiae s.s.* (Ifakara strain) prior to the actual bioassays. A minimum of 20 and a maximum of 25 non-blood female mosquitoes, aged 3–5 days, were exposed for 60 min to the diagnostic concentrations of 0.75% permethrin, 0.05% deltamethrin, or 0.25% pirimiphos-methyl. Similar numbers of mosquitoes were exposed to oil-impregnated papers as controls. Knockdown rates were recorded at 10, 15, 20, 30, 40, 50, and 60 min intervals. After the exposure period, mosquitoes were transferred to holding tubes and maintained on 10% glucose solution. Finally, 24 h post-exposure mortalities were recorded and compared [37].

### Statistical analysis

Data reviews and discussions with the research team were done weekly. Audio data from the in-depth and key informant interviews were transcribed verbatim and then translated from Kiswahili into English. Data were first coded, explored, and interpreted following framework analysis steps described by Gale and colleagues [38]. The transcripts were coded and analysed using MAXQDA® software (VERB; Berlin, Germany) [39, 40]. Codes were generated based on the study questions and through comprehensive and repeated reading of the transcripts. Similar codes were conceptualized, merged, categorized and, finally, developed into themes. Both peculiar and common views supporting themes were observed and recorded. An integration weaving approach was employed, in which both quantitative and qualitative data from the farmers were presented together [41].

Descriptive findings from the quantitative survey were summarized and presented as percentages, and representative direct quotes from different participants are presented to further illustrate the findings. Susceptibility test findings were analysed across the four replicates for each insecticide and percentage mean mortalities, 24-hour post-exposures were interpreted following WHO criteria for interpreting insecticide resistance [37].

## Results

### Demographic characteristics of farmers

Out of 57 farmers who participated in the qualitative in-depth interviews, 30 were females. Of the 420 farmers who participated in the questionnaire survey, there were slightly more males than females (220 vs. 200). The most common crops produced were rice, maize, tomato, vegetables, and fruits for both sale and home consumption. Overall the age of the participants ranged between 21 and 57 years.

### Knowledge and practices related to pests and pest management

The majority of farmers (n = 401, 93.9%) utilized synthetic pesticides in their farms; 374 sprayed herbicides, 285 sprayed insecticides, 66 sprayed fungicides, and 21 sprayed rodenticides on regular basis. The pesticides were mostly used for fear of yield loss and desire to improve productivity. Most of the farmers (70.3%) believed pesticides were effective for pest control. Pesticides were widely subsidized and easily accessible on the local market.

“*In the past, we were buying pesticides only from a big city such as Dar es Salaam, and they were costly, but these times you can purchase most of the pesticides even here in my village*” (male farmer, 53 years).

Pesticides selection and preference were commonly based on experiences and instructions received from the pesticides dealers (Table 2). Overall, slightly fewer than half (n = 198, 46.4%) of the farmers reported being able to identify and name pests on the farms, but most could describe different pests based on their morphological features, including colour, size, and ability to fly. Insects were also described based on the damage they caused (e.g., holes on plant leaves), while some were named using local names in Kiswahili. Weed pests were also described based on physical features, such as soft and hard weeds. Local names were used to identify crop diseases. For example, fungal infections were generally grouped together and referred to as “*Ukungu*”, and bacterial diseases were confused with rust fungal infections on the leaves, and termed as “*Kutu*”.

**Table 2:** Knowledge, practices, and opinions of rural farmers on pest management

Variable assessed	Response	N (%)
Reasons for using synthetic pesticides**	Control pests infestations and improve agriculture production	391 (91.6)
	Easy and effective method to control crop pests and diseases	300 (70.3)
	Increased pests incidence and damage on the crop	21 (4.9)
	Availability of pesticides, subsidized agro input, and initiation of “*kilimo kwanza*” (agriculture first)	123 (28.8)
Ability to identify pests in the farm	Able	198 (46.4)
	Not able	229 (53.6)
Criteria used when selecting and using pesticides	Estimate the size of the farmland	174 (40.7)
	Rely on how extensive the insect pest have infested agricultural land	124 (29)
	Identify the type of weeds (hard and soft weeds)	267 (62.5)
	Spray any pesticide as long as it was effective previously	289 (67.7)
	Others (specify)	26 (6.1)
Awareness of non-chemical pest management methods	Aware	341 (79.9)
	Not aware	86 (20.1)
Use of non-chemical control methods against pests	Yes	153 (35.8)
	No	274 (64.2)
Perceived effectiveness of non-chemical pest management methods	Effective	41 (26.9)
	Not effective	61 (39.6)
	Don’t remember	51 (33.5)
Cultural pest management practices ever used**	Intercropping	178 (41.7)
	Crop rotation	5 (1.2)
	Mulching	6 (1.4)
	Don’t remember	19 (4.4)
	None	234 (54.8)
	Don’t know	23 (5.4)
Awareness of natural enemies/predators for pests control	Aware	102 (23.9)
	Not aware	318 (74.5)
	Don’t know	7 (1.6)
Awareness of integrated pest management	Aware	16 (3.7)
	Not aware	411 (96.3)

*“Kilimo kwanza*” resolution was referred to transformation of agriculture from subsistence into a modern and commercial sector

**Questions with multiple responses options

“*(…). there are insect pests that destroy rice, especially during dry hot season, these pests have hard skin and are black-spotted. They primarily destroy the rice plant by cutting the roots. Unfortunately, I don’t know the specific name of the pest*” (female farmer, 32 years).

Up to 80% of the farmers (n = 341) were uninformed of any non-chemical pest management methods and only a small proportion of those who tried them found such methods effective. Commonly mentioned traditional methods for pest control were use of wood ashes, papaya leaves extract, and a mixture of onion and garlic extract solution against insect pests and fungal infection, as well as mechanical/hand weeding rather than using herbicides. About one quarter (n = 102) knew about using natural enemies for pest control but had never practiced. The majority of farmers (n = 411, 96.3%), had never heard about integrated pest management.

“*I had issues of “finyi” (referred to as caterpillar) in my small Chinese-lettuce garden, I tried to dust wood ashes twice a day and it worked. I will try to use it in my rice farm but I doubt its effectiveness on a large farm*” (female farmer, 28 years).

### Awareness of the associations between malaria transmission, mosquitoes, and agriculture

Most of the participants of the in-depth interviews were knowledgeable about *Anopheles* mosquitoes being vectors of malaria. Some participants knew that there are other mosquito species of medical importance, but they could not differentiate these from malaria vectors. The majority of participants believed malaria mosquitoes breed in stagnant and clean water, however they were unable to link their agricultural activities such as irrigated rice farming (locally known as “*Ngapa*”) to mosquito densities in the agro-ecosystem.

“*I know there are female Anopheles mosquitoes transmitting malaria, but I could not imagine malaria mosquitoes can lay eggs and grow in my rice paddies (locally known as Ngapa)*” (female farmer, aged 27 years).

### Knowledge and opinions regarding effects of agricultural pesticides on malaria vectors

About one third of the farmers had varying opinions on the effects of pesticides on mosquito vectors (Table 3), while the remaining two thirds (65.2%) had no idea on any such effects.

**Table 3:** Farmers’ knowledge, views, and perceived effects of agricultural pesticides in malaria mosquitoes

Variable assessed	Participant responses	N (%)
Opinions about the effect of agricultural chemicals on malaria mosquitoes	Kill malaria mosquito	80 (18.4)
	Chase away malaria mosquitoes	18 (4.1)
	Do not have any effect in malaria mosquitoes	42 (9.7)
	Influence the increase of mosquito population density	10 (2.3)
	Influence insecticide resistance in malaria mosquitoes	1 (0.2)
	I don’t know	283(65.2)
Awareness of insecticide resistance in malaria vectors	Aware	109(25.5)
	Not aware	318 (74.5)
Opinion on what insecticide resistance in malaria vectors means	Increase in mosquito population density	57 (52.3)
	Mosquitoes cannot be killed or repelled by the insecticides	9 (8.3)
	Mosquitoes cause more malaria in the village settings	15 (13.8)
	Mosquitoes are no longer responsive to the insecticidal interventions	11 (10.1)
	I don’t know	10 (9.2)
	Others	7 (6.4)
Opinions on methods to prevent/delay insecticide resistance in malaria vectors	Minimize the use of agricultural chemicals	1 (0.9)
	Minimize the use of public health pesticides against mosquitoes	1 (0.9)
	Establish integrated pest and vector management (IPVM)	2 (1.82)
	Alternative use of biological and environmental methods for controlling pests and mosquitoes	17 (15.6)
	Others	56 (51.4)
	I don’t know	32 (29.4)

Most questions had options for multiple responses

“*I think agricultural pesticides might have an impact on mosquitoes in the farm but I don’t know the details. From my experience, when I use Roundup (glyphosate) chemicals, the surface of the land turns black like rotten materials, perhaps it could upport growth and development of insects such as chamvi (referred to earthworms) and maybe malaria mosquitoes*” (male farmer, 31 years).

Three quarters (n = 318, 74.5%) of the farmers had never heard about insecticide resistance in malaria mosquitoes and could not relate with their use of agricultural pesticides. Among those who had heard of resistance, 35.0% (49/140) believed it meant always having high mosquito population densities in the villages.

“*Insecticide resistance in malaria mosquitoes is a tendency whereby there is always high mosquito population density that means too many mosquitoes each year causing malaria*” (female farmer, 29 years).

### General knowledge of malaria, mosquito biology, and mosquito control methods

Most farmers were informed about malaria and its mode of transmission, and most believed that malaria mosquitoes bite and transmit disease at night (“*usiku wa manane*”). Farmers also claimed that they do experience many mosquito bites during the day, while in the farms and at evening hours when they are cooking, eating, and socializing outdoors, though they were not sure whether these bites were also infectious. Interestingly, none of the farmers could recognize the actual malaria vectors or distinguished them from other mosquitoes. Some believed that malaria vectors are larger than other mosquito species, are coloured, and hide during the day in the bushes then show up at midnight, and hence, challenging to see them.

“*Malaria is a disease transmitted by a special mosquito that bites at 2 a.m. However, honestly speaking, I see lots of mosquitoes moving inside and outside my house, but I cannot identify the mosquito that transmits malaria*” (male farmer, 22 years).

Another male farmer expressed his experiences as follows:

“*I really do not know how the mosquito that transmits malaria looks like. Since they bite at midnight around 2 a.m., it’s difficult to catch, see, and understand the malaria mosquitoes*” (male farmer, 35 years).

A few farmers were also aware of other diseases transmitted by mosquitoes, and frequently mentioned lymphatic filariasis, yellow fever, and dengue.

Bed nets were the most widely used malaria-preventive intervention as reported by the farmers. The most common net brands were those without insecticides (Safi Polyester Bed Net©), purchased from the local stores or the freely-distributed LLINs. In addition, the respondents reported spraying insecticide aerosols indoors during evening hours before bedtime as additional control strategy against hiding mosquitoes that enter houses through open eaves and doors. Other approaches used by a few participants included environmental management (e.g., cleaning the environment, clearing bushes, elimination of breeding sites by filling unwanted ponds, and removing stagnant waters), fanning mosquitoes away with a piece of cloth, dressing babies in long-sleeved clothes, and applying repellent lotions in the evenings when spending time outdoors. Below is some of the responses from farmers.

“*I have been using a bed net against malaria transmission which I purchased from the local shops here in Lupiro, and I do treat with an insecticide called Zuia mbu after every 3 months. But this year we received free bed nets, and we were told they are already treated with chemicals from the industry*” (female farmer, 36 years).

Some farmers who participated in the survey had contrasting responses regarding insecticide resistance in mosquito. Even though they appreciated the benefits of using bed nets, some claimed that the nets, especially those that were freely distributed do not offer enough protection because they have big holes, are of poor texture, are less durable, and are easily stretched after washing. Some farmer also claimed that current bed nets do not have enough chemicals compared to the previous ones, as the mosquitoes could even rest for long periods on them.

“*Even though experts say that the bed nets are impregnated with insecticides still I could find mosquitoes inside the net with blood in their body, in the morning*” (female farmer, 24 years).

“*In the past years bed nets were very heavy, strong, and durable as they could last for years but these days bed nets especially the ones freely distributed are easily stretched and malaria mosquitoes could get in and feed on us*” (male farmer, 44 years).

### Results of the participatory workshops and field visits

#### Farming practices relevant to mosquitoes and their ecology

During the participatory workshops and field visits, farmers explored and learned potential sources of mosquitoes linked to agricultural practices, such as the establishment of rice paddies and irrigation channels. The local practice of irrigating rice fields (i.e., *Ngapa*), and vegetable irrigation in close proximity to river shores were the commonest sources of mosquitoes observed. Most farmlands had small pools and water-filled animal footprints favourable for malaria mosquitoes. Repairing broken rice paddies and maintaining the drainage system were identified as options to reduce mosquito breeding. Other approaches discussed included regular draining and/or replacing water in the flooded rice paddies.

Farmers expressed interests in learning some key morphological features to distinguish between *Anopheles* and other mosquitoes. Using larvae collected from the rice paddies, farmers learned how to distinguish the mosquitoes based on their resting position to the water surface (anopheline larvae rest parallel to the water surface, while culicine larvae rest at an angle). Some of the larvae were raised to adult stage and used for training on how to distinguish *Anopheles* from culicines, using features such as wing spots. Some participants referred to *Aedes* as being the most beautiful mosquitoes (given its black body and white spots). They also referred to male mosquitoes as “bearded” (providing reference to their feathery antennae) just like human males.

“*Ooh! Now I understand that not every mosquito in my house is Anopheles and can transmit malaria, there are other mosquitoes such as Culex which also dominate in our village*” (male farmer, 34 years).

#### Improved knowledge on agricultural pesticides, crop pests, and good agricultural practices

During the visits, it was also observed that farmers were regularly mixing pesticides at the farms, in close proximity to water bodies, and lacked proper disposal of remnant pesticides and empty bottles, which were instead scattered around or emptied into the river waters or irrigation channels. However, the farmers interacted with agricultural experts and learned safe and proper methods for handling, spraying, and disposal of pesticides. They were shown how to read pesticide labels, and how to identify pesticides by both common names and active ingredients. Farmers acknowledged that they had been spraying various pesticides at various dosages based on their experiences without considering potential negative impacts on mosquito ecology and the general ecosystem. Experts also outlined the general safety and management of pesticides and the use of recommended dosages and encouraged farmers to seek additional advice when necessary.

Both farmers and agricultural experts learned the associations between various public health pesticides and agricultural pesticides. The similarities in terms of active ingredients were discussed with the participants. Agricultural experts were concerned with the fact that most of the pesticides available on the market are broad-spectrum and could kill even beneficial insects, which support pollination and some feed on harmful insects.

“*Unfortunately, the majority of the current agricultural pesticides have broad-spectrum/non-selective features and with the limited knowledge among users poses health risks to the community and beneficial insects in the environment*” (male agricultural expert, 28 years).

Extension officers engaged the farmers and scientists in discussion on how to identify crop pests and diseases. Beneficial insects were classified and learned among farmers. With the input from the extension officers, farmers collected crop pests and learned to identify them, and describing diseases affecting vegetables, tomatoes, maize, and rice, best practices for managing pests and diseases in crops, and selection of effective pesticide for a particular crop problem. Among the devastating insect pests and diseases collected and identified, including rice stem borer that infect rice crop, leafhoppers, maize stalk borers, cutworms that infect maize, leaf beetles and aphids that affect beans, spider mite and African bollworms that can infect tomatoes, maize, and vegetables. Common diseases detected in the farms included yellow virus, leaf blight bacterial wilt in tomato farms, leaf rust, and brown leaf spots. Most farmers, however, acknowledged that sometimes they were misidentified and describing the pests using the local names, thus incorrectly apply pesticides. Farmers recommended that the extension officers could create an archive of images of all common pests and diseases on a poster and their corresponding pesticides. Farmers were able to exchange contacts with the experts for further technical support and information on best pest and disease management practices.

#### Results of the insecticide susceptibility bioassays

Researchers briefly described possible effects of agricultural pesticides on mosquitoes, including killing adult mosquitoes and likely influence in the development of insecticide resistance after prolonged use. Insecticide resistance findings were also presented and discussed with the participants. All participants jointly observed the results of the susceptibility tests and were guided through the interpretation.

Following the WHO criteria [37], female adult *An. arabiensis* were found resistant to the two pyrethroids, permethrin (mean 24-hour mortality of 62%) and deltamethrin (mean 24-hour mortality of 60%) and even to the organophosphate, pirimiphos-methyl (mean 24-hour mortality of 58%).

### Feedback from key-informant stakeholders

#### Malaria vector control interventions, benefits and challenges

Most stakeholders acknowledged the overall decline in malaria prevalence over the past 10 years across the country, and attributed this mostly to wide-coverage of LLINs, improved health-seeking behaviour among communities, and improved case management.

“*At least everyone has access to the long lasting insecticidal net, that could have been significantly contributed in malaria cases and deaths reduction, especially in remote areas where health facilities are limited*” (male programme manager, age not disclosed).

However, the ward health officers, who mostly reside in the study area indicated that they had been experiencing high mosquito densities indoors, coupled with an increased in outdoor-biting, making them doubt whether the LLINs still kill mosquitoes.

“*In the past, you will only get bitten at midnight, thus the idea of the government promoting sleeping under an insecticidal bed net, but now even in the evening hours you struggle with the mosquito bites*” (female agricultural expert, 32 years).

#### Linkages between agricultural practices and malaria

Most of the stakeholders were aware of the linkage between agricultural and public health. Some associated transmission risks of diseases, such as malaria and schistosomiasis to agricultural practices. Most of the interviewees identified farming practises that might create aquatic sites for mosquito vectors (e.g., rice flooded paddies).

“*So yes, there is an association between agriculture and health, very clear association and at the moment I’m aware of several initiatives which are trying to bridge between agriculture and health, looking mostly at parasitic infections which are directly linked to agricultural activities, such as malaria and schistosomiasis*” (male director of sciences, age not disclosed).

The interviewees also acknowledged potential effects of agricultural pesticides on mosquitoes. It was argued that continuous use of pesticides could create pressure on malaria vectors breeding in the study area and later facilitate resistance to insecticides. Interviews were also concerned that public health and agricultural sectors still operate independently, each with its own vision. Off-label use of pesticides was identified as common among farmers, due to the lack of awareness and poor communication between agricultural experts and farmers.

“*There are quite some positive and beneficial effects of using agricultural pesticides in crop protection, increasing productivity and some for veterinary purposes. However, there has been little consideration among users on how these pesticides used in agriculture intersects with public health insecticides and have effects in malaria mosquitoes*” (director public sector, 51 years).

#### Controls and regulations relevant to pesticide use

There are multiple laws and regulations in place, overseeing pesticide use in Tanzania, with three different regulatory bodies being responsible. Agricultural pesticides manufacturing, registration, distribution, handling, and usage are governed by the Plant Protection Act no. 13 issued in 1997 [42] and the Plant Protection Regulations of 1999 [43] under the Ministry of Agriculture. In this act “plant protection substances” are referred to as pesticides. The Ministry of Agriculture delegates the Tanzania Plant Health and Pesticides Authority (TPHPA), previously known as Tropical Pesticides Research Institute (TPRI), as the competent authority with the full mandate of registering, approving the quality of pesticides, and licensing of stores selling pesticides. In contrast, Tanzania Food and Drugs Authority (TFDA) law regulates veterinary pesticides.

“*(…) if you look at the law, the law concerning pesticides is fragmented, there used to be a law which was comprehensive and was under the Tropical Pesticide Research Institute Act which it’s part 5 was pesticide control. But then someone came along and gave support to the government saying, “Why are you giving regulatory responsibilities to autonomous institutions, it has to be directly under the government. So the Ministry of Agriculture is the one that is supposed to oversee these regulations.” So they wrote another law on pesticides which they called Plant Protection Substances act in 1997.*” (female senior lecturer public sector, age not disclosed).

“*So when you look at the different laws that touch upon the use of pesticides, they do not provide a very solid border and there is always an overlap of activities, for example in the control of livestock chemicals this should be done by TFDA*” (male senior lecturer at public sector, age not disclosed).

The officials recognized that the majority of distributors and retailers do not comply with the pesticides management laws and regulations, and that there are inadequate pesticides surveillance practices. For example, according to laws, all retailers of agricultural chemicals are required to have a trained and certified by the TPHPA/TPRI prior to the opening of an agrovet store. However, in many cases, licensed agrovet store owners acquire training but do not practice; instead, they employ untrained personnel either a relative or friend to sell the agricultural chemicals. In addition, there are limited numbers of authorised chemicals inspectors appointed by TPHPA/TPRI, and hence, the Ministry of Agriculture allows agricultural extension officers trained by Sokoine University of Agriculture (SUA) and other agricultural bodies to conduct pesticides inspection. Unfortunately, there was also often a conflict of interest, as some of the agricultural extension officers also own agrovet stores.

Illegal importation of substandard pesticides into the market and lack of facilities for adequate disposing of pesticides were also identified as additional challenges. This results in improper use of pesticides and increased the risk of environmental contamination.

“*(…) otherwise we will be flooded with chemicals. But there are few that still come in, because we still have porous boundaries, from Rwanda, Kenya, and Burundi. You find that the regulatory procedures in Kenya are more lenient than ours so other people have found a way to bring in the product through other countries, so you might find product coming in from Rwanda, Burundi, and even from the south coming from Malawi and Zambia coming in as contraband*” (senior staff at public sector, age not disclosed).

However, the issue of sub-standard pesticides could be controlled by using a bar code system.

“*That should be the case for all regulated products especially those we import, there should also be a system for those that we produce in the country, a system of controlling quality and we should find a way to identify that this product is from Tanzania agrochemical producers or suppliers and this is your code, thus creating something which is unique and difficult for people to develop counterfeit. An electronic system for instance if you are in Mpanda district in west Tanzania, you see a product, able to scan, get all the details like QR and others. I should be able to scan using my phone and identify if a product is fake and be able to isolate it. The product information should also include where it comes from*” (director of public sector, age not disclosed).

#### Suggested improvements for pesticides management

The use of a self-surveillance system was suggested as one of the potential approaches to improve management of pesticides usage. There was one example of a successful pilot done in northern Tanzania [44], which established self-surveillance programmes to empower farmers with knowledge and skills to report the pesticide products and quantities and improve decisions on use and dosage.

Mixing of different pesticides was already widely employed by farmers. However, during the programme, the farmers also learned how to select the appropriate pesticides. Farmers become actively involved in observing problems at the farms and reported side effects of pesticides on their health and even ecosystems.

“*What we have been able to pilot is this one tool “self-surveillance system” which was used in Asia, Asia Pacific. It had been used a lot by the Asian people and they were able to help in the banning of pesticides such as Paraquat (paraquat dichloride) and Endosulfan (organochlorine) because they used to record their effects and reported them. After activists picked it up, they blew it into a national issue and the government had to listen and make a decision*” (female senior lecturer at public sector, age not disclosed).

Third was training the trainers who could be ambassadors in the local communities to improve pesticide use. In this study, farmers were able to record, discuss as a team on any side effects associated with the pesticides they have been using, and later decide the way forward to manage the problem in consultation with an agricultural officer.

“*(…) and we tried to train trainers, training a few farmers and letting them go teach others. We taught them how to analyze those forms, conduct calculations, see their effects, and then sit as a group to discuss. They could see that after using large amounts of pesticides many people in their groups would get headaches or feel dizzy so a specific pesticide wasn’t right, they would decide on whether to change it or leave it all together*” (female senior lecturer at public sector, age not disclosed).

Another suggested approach was recycling empty pesticide containers instead of burying, throwing away, or burning. The stakeholders suggested that all leftovers, expired products, or invalid pesticides should be taken to a centralized station following national guidelines, to be collected and dispossed by appropriate authorities. Lastly, barcode systems could help regulate the quality of pesticides coming in the country and control substandard pesticide products.

#### Need for multi-sector collaborations and community empowerment

The key informants acknowledged the need for a holistic approach for integrating relevant sectors in the management of pesticide usage in both public health and agricultural practices. While the main challenge has been implementation costs, and lack of commitment among sectors, possible collaborations could involve pesticides regulatory bodies and the relevant ministries as well as the malaria control programme.

“*But it’s very important to have people from different sectors on issues regarding pesticides because pesticides are used everywhere for different purposes and sometimes are misused not only in the farms but also in the community, some people just use them to kill mosquitoes in their houses, for example, these times, there are fumigation companies everywhere. These companies most likely use the same pesticides approved for agricultural purposes*” (male senior lecturer at public sector, age not disclosed).

In addition, empowerment could start by raising awareness and participatory community involvement on pesticide management and alternative pest control. Open dialogues could be one of the platforms for the stakeholders to meet and hold discussions on the agricultural pesticides usage and its association with public health.

“*Most farmers apply pesticides based on their experience, not sure if they are aware of correct amount and when to spray, so I think, health sector and the agriculture sector need to be interacting at a certain level either through programme interventions or through meetings whereby the open dialogues on the pesticides products and usage practices which seems to be cutting across the two sectors are discussed*” (male director of public sector, age not disclosed).

However, they emphasized that the partnership needs a policy framework that will guide the collaborative approach.

“*In my opinion, integrating only agricultural and public health sectors in pesticides management at the local community level may not be enough. This is because most of these programmes are governed by policy and regulations. Effective implementation programme would require policymakers being part of the game changer*” (director of public sector, age not disclosed).

## Discussion

The use of agricultural pesticides for crop protection is fast increasing in Sub-Saharan Africa, including Tanzania. However, intensive use of agricultural pesticides may influence insecticide resistance in crop pests, cause pest resurgence, and has been linked to other challenges, such as pesticide self-poisoning by farmers [45, 46] and pesticide residue in foods. These chemical residues also accumulate in the aquatic mosquito breeding sites where most of the farming practices are taking place, resulting in a selection pressure on mosquito’s larvae, thus driving the development of insecticide-resistant mosquitoes [47–55].

The current study investigated the knowledge, views, and practices among Tanzanian farmers in two districts about the issue of insecticide resistance in malaria mosquitoes associated to their long-term usage of agricultural pesticides. Opportunities to engage the farming sector in management of insecticide resistance in malaria vectors and crop pests by direct participation in the fields were explored. Most farmers reported pests as a serious challenge to effective crop production, and synthetic pesticides were heavily relied on. The fear of losing crops, subsidies for agrochemical input, and the ready availability of pesticides influenced farmers’ decisions to use pesticides over non-chemical options. While all farmers described pest and disease descriptively, most were not knowledgeable on pest biology, thus pesticides were sprayed haphazardly. Considerable knowledge gaps in pest and diseases identification among farmers have been reported previously [56, 57]. Most of the agricultural insecticides utilized had a broad spectrum, were non-selective, and were indiscriminately sprayed based on farmer experience and informal knowledge received from the sellers [22]. These findings are consistent with those previous study in Tanzania, which found lack of knowledge and poor pesticides usage and disposal practices among farmers and pesticide sellers [44, 58]. A recent performance audit report by the Controller and Auditor General (CAG) of the National Audit of Tanzania (NAOT) highlighted similar issues and recommended actions to be taken to improve knowledge on pesticides handling and agricultural practices among users [20]. It was concluded that there is inadequate implementation of the pesticide laws and regulations governing pesticides management in Tanzania [20, 59].

While most farmers were generally knowledgeable on the link between mosquitoes and malaria, they were not acquainted with the biology and ecology of mosquitoes and their breeding sites. A study by Afrane and colleagues in Ghana found that all farmers who participated in their study had not seen mosquito larvae before and were not aware that water used for agricultural practices support mosquito breeding habitats [60]. In the current study, most farmers were not aware of insecticide resistance in mosquitoes and they could not associate with the selection pressure from pesticides usage. Some farmers claimed the increase in mosquito population density in their localities. *Culex* and *Mansonia* are the predominant mosquito genera in the villages studied here [61–65] that cause biting nuisances and might be perceived as malaria vectors. In the southern part of Côte d’Ivoire, farmers also heavily used agricultural pesticides, while they were unaware of the threat to develop insecticide resistance in mosquito vectors [66]. The lack of awareness of mosquito ecology and biology and the concept of insecticide resistance in mosquito among farmers could have negative implications when designing and implementing vector control programmes. The study recommends regular educational programmes, including community engagement sessions and involvement in the research activities and malaria vector control programmes, in line with previous experiences and recommendations [67–69].

Over 96% of farmers indicated that they had never heard about the concept of integrated pest management (IPM), although a third had previously implemented some non-chemical pest control practices, which are among components of IPM programmes. The alternative traditional pest control methods to synthetic pesticides include the use of wood ash and manual weeding [70]. However, farmers did not routinely use non-chemical pest control methods, as these methods were considered less effective compared to pesticides, and could not be deployed in a large farm-scale because of the fear of reduced crop yields due high incidence of pest infestations. Intercropping farming practice (e.g., maize intercropped with beans, or maize intercropped with sesame) was routinely implemented by 42 farmers. This strategy was primarily considered as a means of maximizing the use of land in order to increase crop yields rather than IPM. Similarly, bean farmers in Tanzania were not aware of other benefits of intercropping, in addition to enhancing the productivity of the farmland [71]. In the present study, 75% of farmers were not familiar with biological/natural pest enemies. Previous studies have shown that these cultural practices such as intercropping could encourage predator biodiversity and reduce the incidence of crop pests, while minimizing the need for using synthetic pesticides [71].

There are several successful approaches for engaging communities against malaria transmission in malaria endemic settings [72]. This study explored possible ways to engage and empower farmers through participatory workshops and practical sessions with the farmers. The study provided a forum for the health researchers and agricultural experts to discuss, interact, actively engage, and empower farming communities with basic knowledge and skills on malaria issues, crop pests, pesticides management, and general good agricultural practices. Improved agricultural practices, including improved management of agricultural pesticides may contribute in preventing/delaying insecticide resistance in both mosquito vectors and pest crops. This approach was based on knowledge sharing and learning practices by researchers who promote awareness among farmers on the linkage between malaria and agriculture, insecticide resistance in malaria vectors and pest crops, and collective resistance management strategies through directly empowering farmers, enhanced with agricultural experts. Indeed, farmers were offered an opportunity to interact with agricultural experts and researchers. The study focused on empowering farmers with basic knowledge and skills on good agricultural practices, including agrochemicals management, which in turn could indirectly minimize the odds of insecticide resistance development in malaria vectors.

Participatory workshops and actively involving the community in mosquito control has been reported previously [73, 74], and could improve uptake of research outcomes into the communities [75], enhance their knowledge on agro-ecosystem practices linked with malaria, while empowering them to make sound agricultural decisions. Feedback sessions with the community and other relevant stakeholders encourage sharing of research findings that could initiate policy changes. As a logical next step, an engagement study with the farmers, researchers, public health, and agricultural experts through workshops and practical field sessions is indicated. Stories should be shared on morphological recognition of different mosquito species, including *Anopheles* and *Culex* mosquitoes, insecticide resistance in mosquitoes, and potential associations with the overwhelming use of agricultural pesticides. Discussions with the farmers on resistance management include proper usage, storage, disposal of agricultural pesticides, and alternative crop pests control other than using agricultural pesticides to prevent or delay the development of insecticide resistance crop pests and malaria mosquitoes.

The majority of the stakeholders acknowledged that agricultural practices have significant implications in malaria vector control. They are aware that pesticide usage practices could be the root cause of insecticide resistance in vectors. They did, however, warn that pesticides usage for crop protection cannot be fully avoided, but must be minimized, used sparingly, or integrated with non-chemical methods. They noted that there is a gap in linking agriculture and public health, probably due to limited resources, such as a shared budget for implementation and lack of commitment across the sectors. While an integrated vector management (IVM) concept for malaria control is encouraged and has been promoted in other East African countries, it is not optimally implemented due to shortage of financial resources and poor implementation approaches [76]. The majority of stakeholders advocated for public forum and field-based farmer field school learning programmes to raise awareness and active participation of communities, policy, and decision-makers. Existing National malaria control strategies could adopt and customize the WHO integrated vector and pest management (IVPM) policy framework in collaboration with key stakeholders from other sectors for a successful malaria vector control programme.

To sustain the effectiveness and efficacy of vector control interventions, the WHO recommends IVM strategies that encourages collaborative efforts within the health sector and across other sectors [30, 31]. Besides, the Food and Agriculture Organization (FAO) of the United Nations promotes combined pest management approach to reduce pesticides application, through IPM farmer field schools programmes [77]. The integrated strategy has the potential to bridge the gap between agriculture and health; nevertheless, it is underutilized in low- and middle-income countries [78]. In Tanzania, the concept of IPM is adopted in the pesticides regulatory policy [42, 79], but its implementation in the communities is limited due to lack of awareness, a top-down delivery, and the widely available and heavy use of subsides as agrochemical inputs. IPVM approaches engage and empower farmers in controlling crop pests, mosquito densities, and malaria prevalence [16].

Due to financial and time constraints, the current study only covered four discussion workshops and three learning-by-doing sessions in the field unlikely a typical farmer field school. With the limited budget, the study did not monitor and evaluate the effect of community-based participatory workshops and fields training on the improved agro-ecosystem practices linked to mosquito and malaria among participants. Hence, future studies should also monitor the impact of participatory workshops on improved knowledge and skills among farmers and other key stakeholders.

## Conclusion

Farmers had some general knowledge that mosquitoes exist in their surroundings and they could associate with malaria transmission, but they could not distinguish malaria from non-malaria vectors. Farmers could not link agricultural pesticides use and insecticide resistance in malaria vectors. Both pyrethroids and organophosphate are used in public health, which were also found extensively used by the farmers. The creation of awareness among the farming community about malaria vectors, the use of agricultural pesticides, and the likelihood of influencing insecticide resistance in malaria vectors is critical in integrated insecticide resistance management strategies in malaria mosquitoes and agricultural pests. For successful pesticide resistance control in mosquito and crop pests, regular community participation, advocacy, and integrating programmes across researchers, public health, and agricultural sectors are required.

## Data Availability

The raw datasets used and/or analysed during the current study are available from the corresponding author on reasonable request.

## Abbreviations

LLINS: Long-lasting insecticidal nets
IRS: Indoor residual spraying
WHO: World Health Organization
TPHPA: Tanzania Plant Health and Pesticides Authority
TPRI: Tropical Pesticides Research Institute
IVM: Integrated vector management
IPM: Integrated pest management
IPVM: Integrated vector and pest management
